# Multipolar-sensitive engineering of magnetic dipole spontaneous emission with a dielectric nanoresonator antenna

**DOI:** 10.1038/s41598-021-92322-9

**Published:** 2021-06-17

**Authors:** Mojtaba Karimi Habil, Carlos J. Zapata–Rodríguez, Mauro Cuevas, Samad Roshan Entezar

**Affiliations:** 1 Faculty of Physics, University of Tabriz, 51664 Tabriz, Iran; 2 Department of Optics and Optometry and Vision Sciences, University of Valencia, Dr. Moliner 50, 46100 Burjassot, Spain; 3 Consejo Nacional de Investigaciones Científicas y Técnicas (CONICET), Buenos Aires, Argentina; 4 Facultad de Ingeniería, Universidad Austral, Mariano Acosta 1611, Pilar, Buenos Aires Argentina

**Keywords:** Optics and photonics, Sub-wavelength optics

## Abstract

We propose an axisymmetric silicon nanoresonator with designed tapered angle well for the extraordinary enhancement of the decay rate of magnetic dipole (MD) emitters. Due to the resonant coupling of a MD emitter and the MD mode of the subwavelength resonator, the Purcell factor (PF) can easily reach 500, which is significantly higher than the PF when using a silicon nanosphere of the same size. The PF and the resonance frequency are conveniently tuned through the resonator diameter and the taper angle of the blind hole. When supported by a metallic substrate, further enhancement ($>10^3$) of the MD spontaneous emission is triggered by an image-induced quadrupolar high-*Q* mode of the nanoantenna. For the sake of comparison we include a critical analysis of the canonical problem that considers a Si spherical shell. Our results might facilitate a novel strategy for promising realizations of chip-scale nanophotonic applications.

## Introduction

Modification of the spontaneous emission rate of an emitter induced by the interaction with its environment has been referred to as the Purcell effect^1–3^. Customization of electromagnetic (EM) radiation of an electric dipole (ED) and MD, devoted many studies during the past decades, is highly desired for many state-of-the art applications such as sub-wavelength semiconductor lasers^4^, electro- and magneto-responsive nanostructures for control of single emitter luminescence^5^, single-molecule optical microscopy^6,7^, and on-chip integrated systems for information processing and quantum communications^8^. The interaction of the EM waves with photonic materials is often of electric nature due to the strong coupling of the electric field and the induced ED in matter. Therefore, an enormous number of studies have been focused on the enhancement of the ED decay rate. Moreover, due to the weak coupling of MD modes of materials and optical waves, the effect of the MD emission has been overlooked.

The progress in nanofabrication and the advent of magnetic quantum emitters, such as rare-earth ions^9^ and semiconductor quantum dots^10^, has stimulated the investigation of the MD Purcell effect. By locally increasing the strength of the magnetic field to some orders of magnitude, the magnetic field and MD mode coupling becomes significant. When a small resonator is excited by an EM wave, inherent localization of the magnetic field creates a magnetic hot spot in the inner part of the resonator. Although, this hot spot can be exploited in some experimental designs such as buried quantum dots^11^, to access the magnetic hot spot and utilize it effectively, manufactured hollow structures in different shapes have been proposed. Some structures, including nanophotonic structures^12^, metallic nanocap^13^, tapered hollow hyperbolic metamaterial^14^, and split ball resonators^15^ have been designed to enhance the emission rate and scattering efficiency of MD emitters.

Over the last few years, some researches have been focused on silicon hollow nanocavities, with special emphasis in silicon cylinders with an air coaxial void, which can maximize the magnetic field in the cavity hole. These structures potentially enhance the Purcell factor in two orders of magnitude^16–19^. Meanwhile, high index dielectric nanostructures having inherently strong Mie resonances at optical frequencies with minimal dissipative absorption are of interest for MD radiation enhancement. In such a way, a MD mode can be excited in silicon nanospheres and silicon nanodimer from visible to infrared spectrum^20–23^. Some other complex structures, including hollow spoof plasmonic structures, constructed of periodically metallic strips in a hollow silicon cylinder, can significantly enhance the quality factor of the magnetic resonance and MD emission^24,25^. In the context of light-matter interaction within the near-field, a variety of tapered nanoantennas^26–28^ and tapered grove nanostructures^29–31^ demonstrate focusing a strong EM field at the tip side.

From a practical point of view, considering that emitters and nanostructures are typically supported by a flat substrate, it has been shown that complex substrates can also be engineered to further manipulate the optical responses of the system^32^. In particular, dielectric nanoparticles supported by a metallic substrate without the need for a high-quality external cavity can excite different resonances efficiently^33^. Moreover, new resonant modes originated from the coherent interaction between the multipole modes of the dielectric nanoparticles and their mirror images induced by the metal substrate can bring dramatic line-width compression and enhancement of the Purcell factor^34–37^.

Here, we analyze dielectric spherical nanoresonators with drilled axisymmetric conical hole. The proposed silicon resonators are efficiently excited by a MD emitter set inside the hole. The effects of the resonator size, the point-dipole location inside the resonator, and the opening angle of the conical hole are analyzed exhaustively. It is evidenced how the PF and the resonance frequency severely depend on the geometry of the resonator. Our results are compared with those analytically obtained for silicon spherical cavities with a void hole. Finally, the substrate effect on the PF and the resonance frequency of the optical cavity are studied, when the resonator is supported by a metallic substrate. In this case, the mirror-induced resonator enables a dipole-dipole coupling resulting in a semi-dark mode, however boosting the magnetic PF through a high-*Q*-factor magnetic-quadrupole mode of the resonant structure.

## Results and discussion

**Figure 1:**
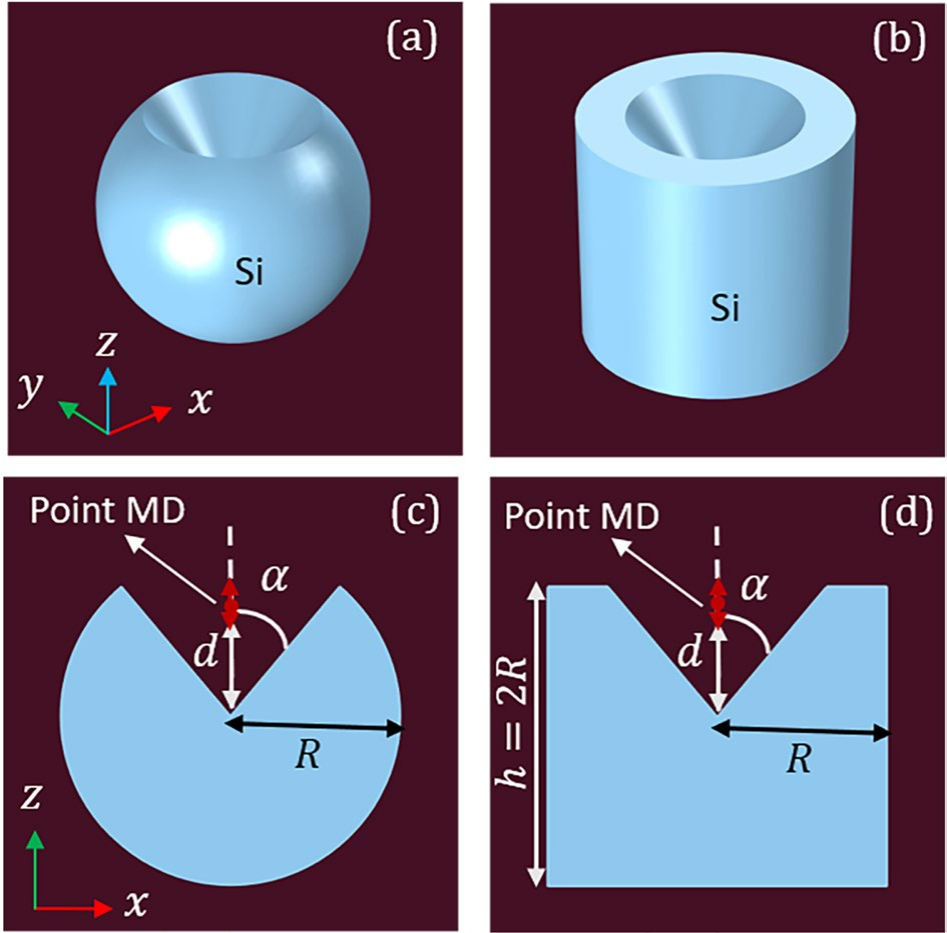
Illustration of (**a**) spherical and (**b**) cylindrical silicon resonators of radius *R*. The cylindrical body has a heigh $h = 2R$. A conical well with angle $\alpha$ and centered apex is additionally drilled in the sphere and cylinder. Note that the well apex lies at the origin of the transverse plane $z=0$. In (**c**) and (**d**) we represent a meridional section (*xz*-plane) of the solid of revolution shown in (**a**) and (**b**), respectively. A MD point source is set in the *z*-axis of revolution, also oscillating along the *z* direction, which is inserted in the tapered angle well at a distance *d* from the center of the resonators.

We start our analysis with the following proposed silicon structures: a sphere of radius *R*, and a cylinder with the same radius and height $h = 2R$. In both cases, a coaxial cone-shaped hole having an opening angle $\alpha$ is practiced on the bodies such that its apex is centered at the origin of coordinates, as schematically shown in Fig. 1. The surrounding medium of the resonators is assumed to be air of unit refractive index. In addition, a point MD oscillating in the *z* direction is located on axis, inside the conical hole, at the distance *d* from the center of symmetry (that is the origin of coordinates) of the resonator. For the sake of clarity, the geometrical parameters in the intersectional *xz*-plane of Fig. 1a,b are shown in Fig. 1c,d, respectively. Both resonators are made of silicon having a dispersive index of refraction in the optical spectra as taken from Ref.^38^.

**Figure 2:**
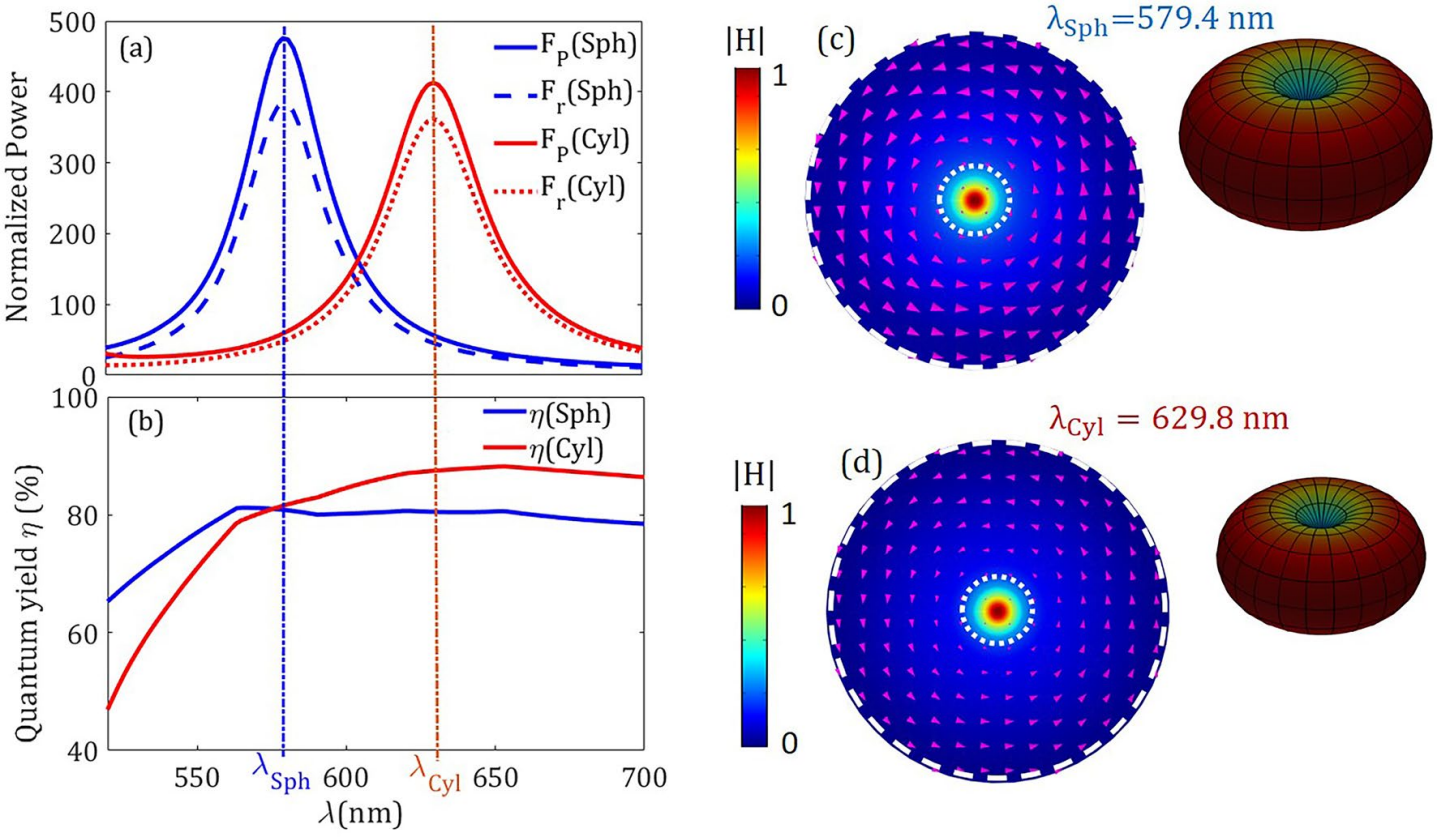
(**a**) Purcell factor $\text{ F}_\text{ P }$, normalized radiated power $\text{ F}_\text{ r }$, and (**b**) quantum yield $\eta$ for the resonators in Fig. 1a,b denoted by Sph and Cyl, respectively. Also, field magnitude $|\text{ H } |$ in arbitrary units for the corresponding resonance wavelengths (**c**) $\lambda _\text {Sph}$ and (**d**) $\lambda _\text {Cyl}$ of the analyzed resonators are shown in the *xy*-plane with $z = 20$ nm. The arrows show the electric vector field, and the dotted and dashed white circumferences show the boundaries of the hole and the resonator, respectively. The magnitude of the magnetic field in the far-field is shown in the inset. Due to the higher radiated power at the resonance wavelength for the case of the spherical resonator, its doughnut-shaped far-field pattern appears larger when compared with the cylindrical resonator. Here, the geometrical parameters are $d=10$ nm, $R=70$ nm, and $\alpha =30^ \circ$.

Firstly, we examine the effect of the presence of the resonator on the decay rate of the inner MD emitter. Figure 2 shows the spectrum of the Purcell factor $\text{ F}_\text{ P }$ and the normalized radiated power $\text{ F}_\text{ r }$ (see “Methods” section) for the silicon nanoresonators described in Fig. 1a,b. As illustrated in Fig. 2a, the spectra of the magnetic PF as well as the normalized radiation power present some similarities when using either the silicon cylinder or the sphere. However, the resonant frequencies that are characteristic of each type of scatterer are slightly different. More specifically, the resonances of the holey spherical and cylindrical resonators occur at $\lambda _{\text{ Sph }}=579.4$ nm and $\lambda _{\text{ Cyl }}=629.8$ nm with PFs attaining $\text{ F}_\text{ P }\text {(Sph)}=476$ and $\text{ F}_\text{ P }\text {(Cyl)}=413$, respectively. These values of the PF presented here are notably higher than others previously reported when considering for instance cylindrical holes as in^17^; namely a PF increment of 36 % and 18 % in the spherical and cylindrical resonators, respectively. In both resonators, additionally, a significant part of the emitted power is radiative in nature thus reaching a high quantum efficiency with the values $\eta \text {(Sph)}=81\%$ and $\eta \text {(Cyl)}=88\%$. Note that the cylindrical resonator gains a slightly higher quantum efficiency at its resonance frequency, as shown in Fig. 2b, which is largely caused by the lower absorption of silicon at decreasing frequencies in the visible.

To illustrate the magnetic dipolar behavior and the radiation enhancement of the emitted light, Fig. 2c,d show the magnetic field amplitude and the electric vector field lying in the *xy*-plane with $z = 20$ nm at the resonance wavelength that is characteristic of each silicon resonator. Note that the near field is represented in the plane transverse to the *z*-axis set 10 nm above the point emitter. The magnetic field is strongly localized in the conical well, whereas the electric field spins around the point emitter mainly centrifuged inside the silicon resonator. In fact, the electric field at points lying on the hole is much lower than that evaluated inside the silicon body.

We further analyze the angular distribution of the field radiated by the emitter. In particular, the magnitude of the magnetic field is depicted in the inset of Fig. 2c,d when evaluated in the far-field at the resonance frequency of each geometry. In both cases, the far-field has the well-known doughnut-shaped pattern for a MD moment oriented along the *z* direction. Moreover, the spherical-resonator pattern is larger than the cylindrical one indicating a higher radiative energy at resonance for the case of the spherical resonator in comparison with the silicon cylinder. Due to the fact that the EM response for both spherical and cylindrical resonators present roughly the same behavior, mainly differing in the peak value of its PF that is always larger for the sphere, from here on all the calculations are shown for the spherical resonators exclusively.

In order to gain insight about the correct interpretation of the spectral peaks in the Purcell enhancement shown in Fig. 2a, the scattered EM field is set in the form of a field expansion in spherical vector waves. This procedure allows us to estimate the effective contribution of each natural EM mode supported by the silicon resonator to the total power scattered by the MD emitter, which is placed inside the conical well. For that purpose it is necessary to apply the multipoles decomposition method^39,40^, thus estimating the scattering coefficients of the electric $a^{(E)}_{l,m}$ and magnetic $a^{(M)}_{l,m}$ multipoles, which are given by (see Supplementary Information)

1 $$\begin{aligned} a^{(E)}_{l,m}= & {} -\frac{k}{Z_0 h_l^{(1)}(kr)\sqrt{l(l+1)}} \int Y^*_{l,m} {\mathbf {r}}.{\mathbf {E}} \, \text {d}\varOmega , \end{aligned}$$

2 $$\begin{aligned} a^{(M)}_{l,m} = \frac{k}{h_l^{(1)}(kr)\sqrt{l(l+1)}} \int Y^*_{l,m} {\mathbf {r}}.{\mathbf {H}} \, \text {d}\varOmega , \end{aligned}$$

where *k* and $Z_0$ are the wavenumber and wave impedance in free space, whereas $h_l^{(1)}$ and $Y_{l,m}$ are the Hankel function of the first kind and the scalar spherical harmonic, respectively. Also, *l* and *m* are the polar and azimuthal modal numbers, respectively, characterizing the multipolar geometry. In Eqs. (1) and (2), the integral runs over the total solid angle of a hypothetical sphere of radius *r* enclosing the resonator and emitter. Using these scattering coefficients, we can establish the coupling strength between the point emitter and the electric/magnetic dipole (ED/MD) modes occurring at $l = 1$, and the electric/magnetic quadrupole (EQ/MQ) modes ($l = 2$) of the resonator, as representants of the lowest-order natural modes of the silicon resonator. More specifically, the contribution of each mode to the radiative power is written as^41^

3 $$\begin{aligned} P^{(E,M)}_{l,m} = \frac{Z_0}{2k^2} \left| a^{(E,M)}_{l,m} \right| ^2 . \end{aligned}$$

Note that for a MD emitter set along the axis of symmetry of a scattering body, only magnetic modes (or TE-polarized modes satisfying ${\mathbf {r}}.{\mathbf {E}} = 0$) with $m = 0$ can be excited (see Supplementary Information). In conclusion, the scattering coefficients set in Eqs. (1) and (2) enable a numerical procedure to estimate the contribution of each one of the EM modes to the spontaneous emission.

**Figure 3:**
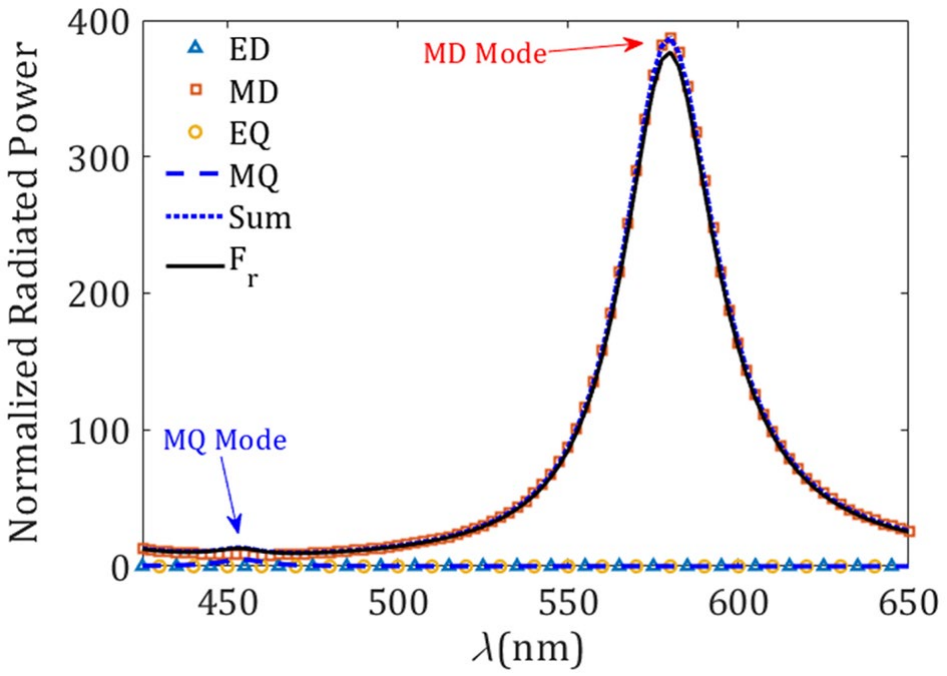
Modal radiative power as given in Eq. (3), normalized to the free-space radiation power $\text{ P}_0$, corresponding to the lowest-order modes excited in the spherical silicon resonator with $R = 70$ nm excited by a MD emitter. Other geometrical parameters are $d = 10$ nm and $\alpha = 30^\circ$.

In Fig. 3 we show the normalized radiated power as previously depicted in Fig. 2a, here conveniently decomposed into its different EM modes. The geometrical parameters of the spherical silicon resonator are $R = 70$ nm and $\alpha = 30^\circ$, and the MD emitter is set at a distance $d = 10$ nm from its center. For the sake of comparison, we normalize the radiated power of each mode, as given in Eq. (3), to that of an isolated MD emitter. As expected, the ED and EQ modes has no contribution in the radiated power, while the radiation of the point emitter is mainly coupled to the MD and MQ modes of the resonator. Moreover, the radiated power of the MD emitter is enhanced at the wavelengths $\lambda =580$ nm and $\lambda =454$ nm corresponding to the MD and MQ natural modes of the resonator, respectively. This fact proves the dominant MD and, to a notably lesser extent, MQ Purcell enhancement for the longest and shortest wavelength, respectively. Finally, by summing the normalized radiated power of every multipolar mode up to the quadrupolar order (see dotted line) and comparing this result with the total radiated power $\text{ F}_\text{ r }$ (solid line) numerically estimated as outlined in the “Methods” section, it is evident the negligible contribution of higher-order EM modes.

**Figure 4:**
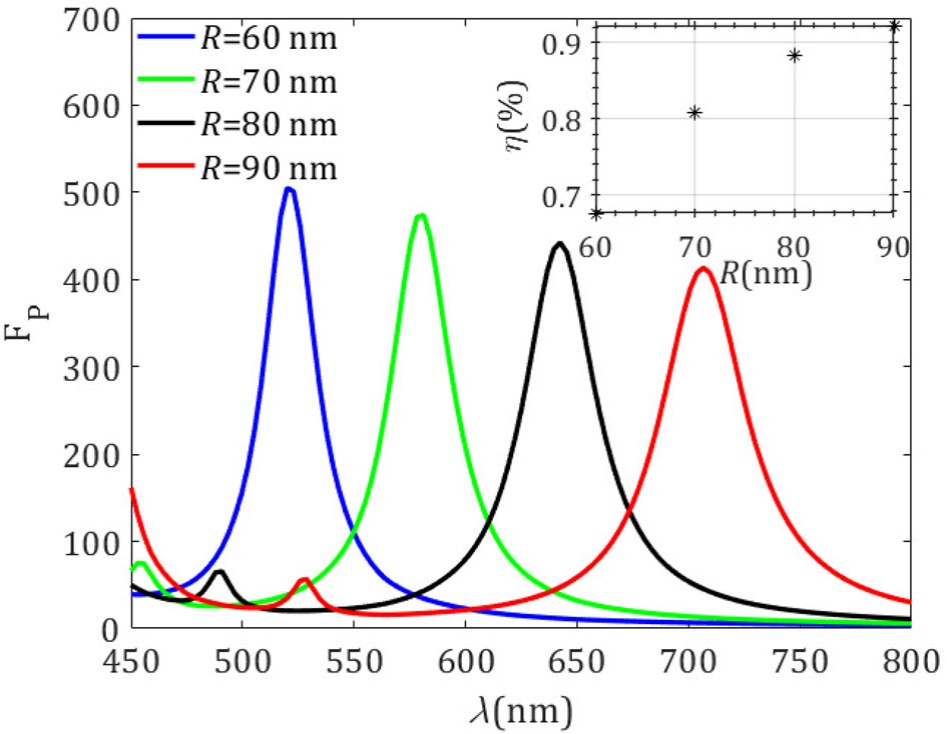
Purcell factor $\text{ F}_\text{ P }$ for different radii *R* of the silicon spherical resonator. The fixed geometrical parameters are $d=10$ nm and $\alpha =30^\circ$ as in Figs. 2 and 3. Inset: quantum efficiency $\eta$ versus *R* at peak frequencies.

Figure 4 shows the PF within the visible regime for different radii of the silicon spherical resonator. An angle $\alpha =30^\circ$ well is drilled, and a MD emitter is set at a distance $d = 10$ nm from its apex. By decreasing the volume of the silicon resonator, the PF increases and the dominant MD resonance peak is blue-shifted. In fact, the MD resonance wavelength approximately satisfies the relationship $\lambda = R/n$, where *n* is the refractive index of the sphere^22^. Such dependence of the PF peaks with the size of the resonator enables a potential tunability of its spectral response. Importantly, the Purcell factor for the resonator of radius $R=60$ nm slightly surpasses a value of 500, which is much higher than other PFs in silicon cavities of similar characteristics^22^. Moreover, the larger size of the resonator the higher quantum efficiency, as shown in the inset in Fig. 4, which arises from the low loss of silicon in the long-wavelength range. In addition, we observe small peaks for low wavelengths, caused by excitation of MQ modes, of particular relevance for resonators of larger radii.

At this point, it is important to note that a similar spectral behavior of the PF can be found for instance in silicon spherical resonators with a centered air hole. The latter problem can be treated analytically, as discussed in the Supplementary Information. For instance, the emergence of a strong MD resonance and a weak MQ peak in the spectral response of the PF, with a clear redshift at increasing volume of the silicon shell can be observed in Fig. S2a–c. Furthermore, the quality factor *Q* for the MD mode decreases with the increment of the outer radius in the shell resonator, as shown in Fig. S2d, a fact that provides the drop of the Purcell factor. The similarity between features of both structures, i.e., between the spherical hollow cavity and the silicon sphere with a cone well, suggests that the same *Q* reduction takes place for these last resonators, leading to a decrease in the PF as shown in Fig. 4.

**Figure 5:**
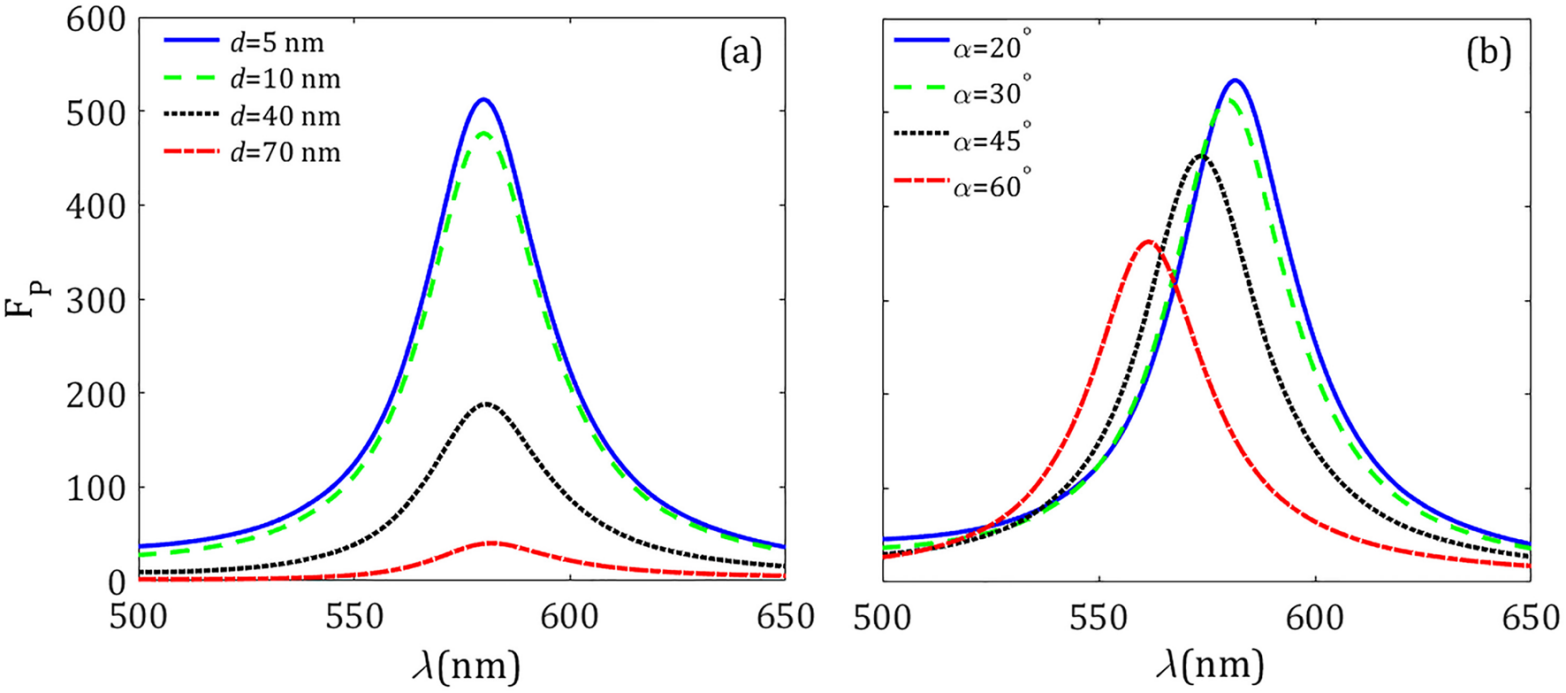
Purcell factor $\text{ F}_\text{ P }$ (**a**) for a different displacement *d* of the MD emitter along the axis of symmetry of the resonator, keeping $\alpha =30^ \circ$ and $R = 70$ nm fixed, and (**b**) for different angles of the tapered well, provided that $d = 5$ nm and $R = 70$ nm.

Next we analyze the dependence of the decay-rate enhancement with the position of the MD emitter and the cone angle of the silicon resonator. In Fig. 5a we show the PF spectrum for several values of the distance *d* to the center of the sphere while the opening angle $\alpha = 30^ \circ$. We observe that the further the MD is shifted from the center of resonator the lower Purcell factor. It occurs in a manner that the PF drops significantly when the magnetic dipole is one radius ($d = R = 70$ nm) away from the resonator center. On the other hand, Fig. 5b shows the Purcell factor spectra for various values of the opening angle, $\alpha =20^\circ , 30^\circ ,45^\circ$ and $60^\circ$, keeping the distance $d = 5$ nm fixed. By increasing the opening angle of the cone, i.e., by increasing the volume of the air well, a blue shift of the resonance wavelength takes place. Most importantly, the Purcell factor is significantly reduced with the opening angle $\alpha$ of the cone well. As can be observed from our previous discussion on the near field pattern shown in Fig. 2, the magnetic field is strongly localized in the conical hole, thus by increasing the hole size the magnetic field is confined in a larger volume, which reveals that the quality factor of the MD mode of the resonator will decrease with the angle $\alpha$; ultimately such behavior is governed by the material dispersion of silicon. Finally, an analogous blue shift and PF drop is observed in cylindrical nanocavities^42^ and spherical hollow cavities (see Fig. S3a), when the hole volume is increased.

**Figure 6:**
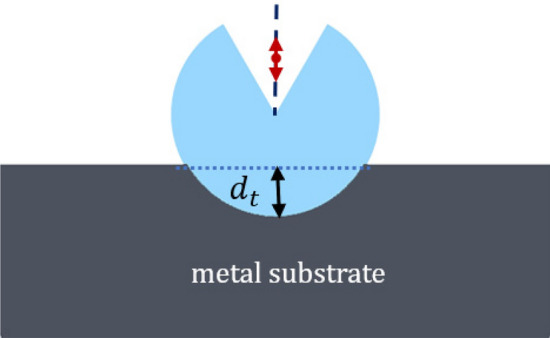
The silicon spherical resonator of Fig. 1 as loaded on the metal substrate. The substrate sag outlined by the overlying resonator body is characterized by the depth $d_ t$. For numerical purposes we consider a silver substrate.

### Metal substrate boosting the magnetic Purcell factor

A thorough analysis of the effects on the magnetic PF concerning the presence of a flat metallic substrate sustaining our holey spherical resonator is presented below. This scheme is of interest not only in a practical implementation of our proposal, but also as a procedure to boost the magnetic PF. Here we consider the resonator is partially embedded on the metallic substrate as shown in Fig. 6, characterized by the depth parameter $d_t$, even conserving the axial symmetry of the original problem. The substrate material is assumed to be silver with a dielectric constant taken from Ref.^43^ for numerical purposes.

**Figure 7:**
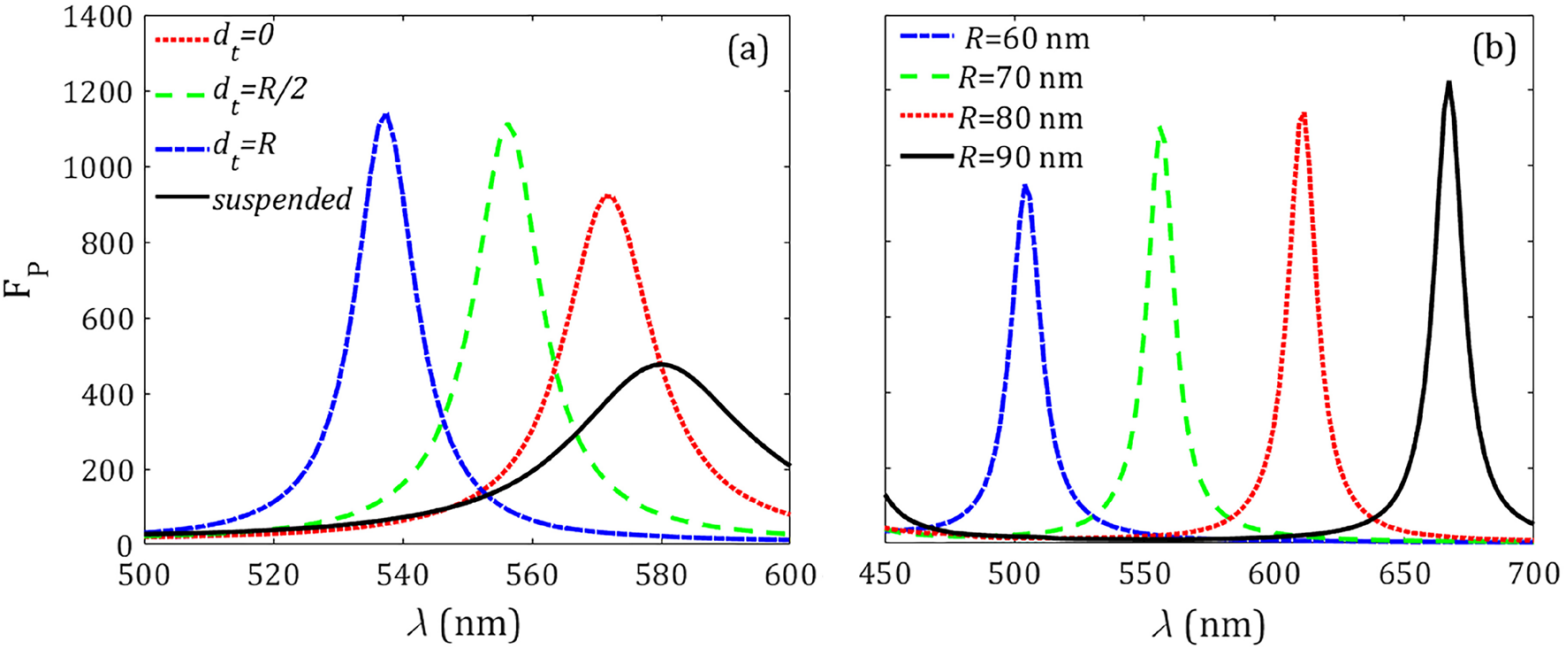
(**a**) Purcell factor of the structure shown in Fig. 6 for different $d_t$, fixing the emitter location at $d=10$ nm from the resonator center, $R=70$ nm, and $\alpha =30^\circ$. For comparison, the PF of the dipole in the absence of substrate with the same parameters has been included (solid curve). (**b**) Purcell factor for different resonator radii while keeping $d_t = R/2$.

In order to gain insight into the modification of the decay rate induced by the metal substrate, in Fig. 7, the magnetic PF is estimated for different configurations, including when the resonator is in contact at a single point of the planar substrate and when it is fully embedded in the substrate. A strong enhancement of the peak PF occurs when the *suspended* resonator in free space is immediately placed at point contact ($d_t = 0$) with the metallic substrate, as shown in Fig. 7a. In this case, the peak wavelength is slightly blue-shifted, a response that remarkably occurs in opposition to the case of EM emitters. When the resonator is further embedded into the metallic medium, the peak PF grows moderately. Moreover, a significantly enhanced blue-shift is observed in Fig. 7a as governed by the penetration depth $d_t$. An increment of the *Q* factor of the resonance is also evident by the progressive narrowing of the PF peaks as long as the silicon body deepens at higher values of $d_t$. The metallization of the resonator walls enables the significant reduction of radiative losses thus moderately enhancing the decay rate of the emitter set inside.

Next we examine the magnetic PF of half-embedded ($d_t = R/2$) silicon resonators with different sizes, shown in Fig. 7b. By increasing the radius *R* of the spherical resonator, the peak PF grows monotonically as long as its resonance wavelength undergoes a remarkable red-shift. As previously examined in Fig. 4, such spectral shift occurs in parallel trend when compared with a resonator suspended in free space, as expected. On the other hand, here, a larger resonator enables an enhanced decay rate of the MD emitter. Interestingly, partial metallization of the resonator walls allows the nanoantenna to support resonant modes with enhanced quality factor when *R* increases, validating the improvement of the peak PF.

**Figure 8:**
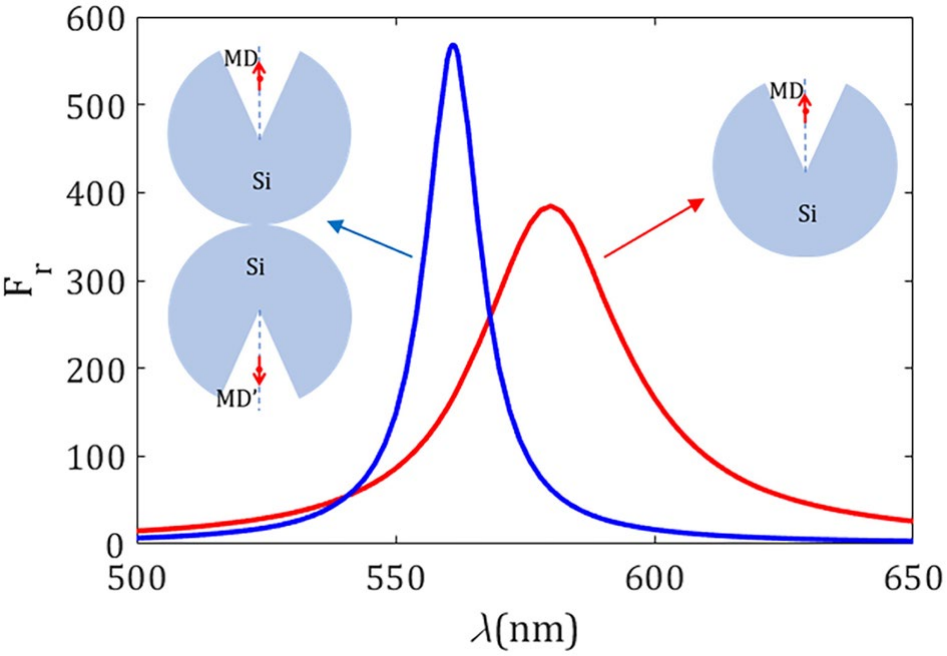
Normalized radiated power $\text{ F}_\text{ r }$ for the suspended resonator in a vacuum (red line) excited by a MD emitter. Also radiated power for the resonator coupled to its mirror image (blue line) is included. The radius of the resonator, its opening angle, and the dipole distance from the sphere center are $R=70$ nm, $\alpha =30^\circ$, and $d=10$ nm, respectively.

The observed increase of the peak PF and its inherent blue-shift can be interpreted in terms of the image theory. For simplicity we will deal with a dielectric resonator at point contact ($d_t = 0$) with the metallic substrate. On that basis, that is valid qualitatively in the optical regime, one may substitute our silver mirror by a perfect electric conductor (PEC). In such scheme we equivalently introduce an image source, which in our case includes an image silicon resonator in order to reproduce the EM fields in the half space above the PEC. Unlike the case of an ED point source set near a PEC, the magnetic dipole and its corresponding image MD have opposed directions. Furthermore, note that when an isolated MD is set extremely close to a flat PEC, a cancellation of the radiated power and thus a drop in the magnetic PF is expected.

Figure 8 shows the normalized radiated power $\text{ F}_\text{ r }$ for the resonator on the PEC, as evaluated under the image-theory approach and, for the sake of comparison, that for the suspended resonator in a vacuum. The effect of the presence of the PEC mirror is a blue-shifted resonance with radiation peak enhancement. Note that this fact is partially counter-intuitive since the interaction of two out-of-phase close MDs is expected to drop the total radiated power significantly. As a consequence, the presence of the silicon resonator near the PEC has a crucial contribution is this effect.

**Figure 9:**
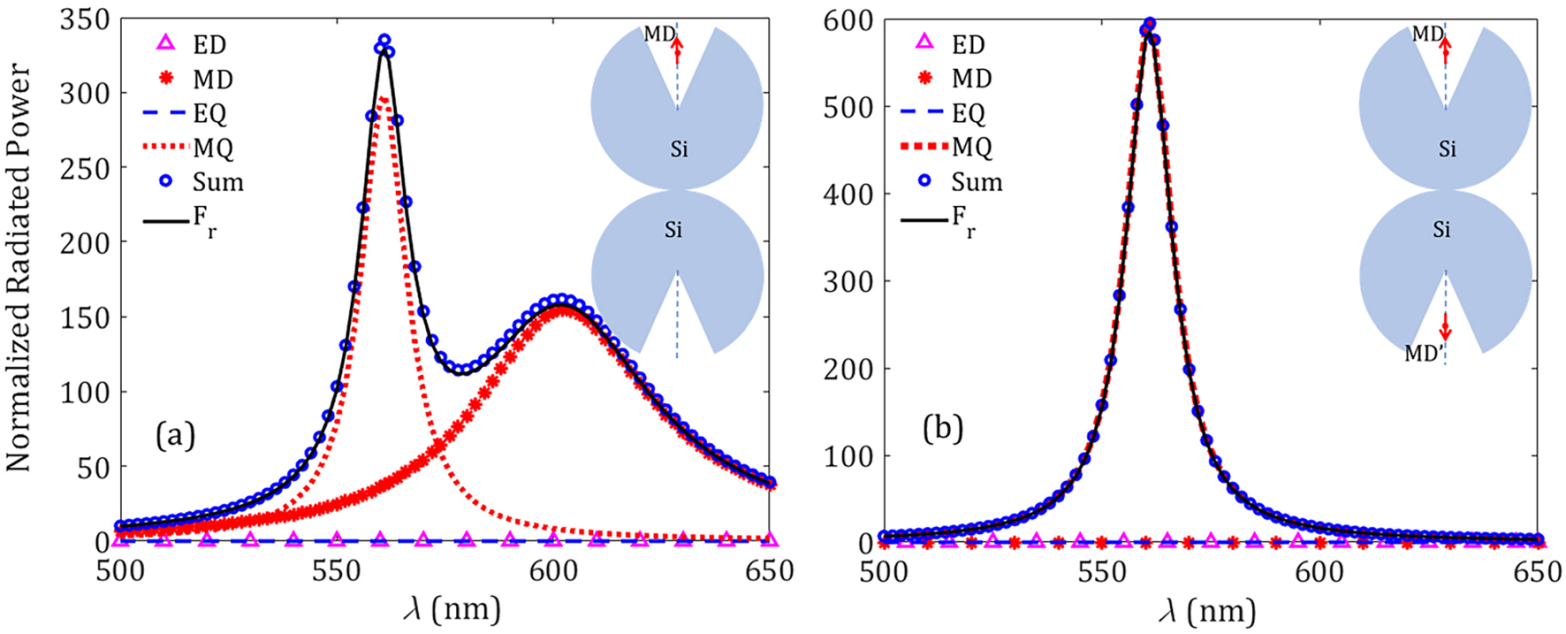
Modal radiative power, corresponding to the lowest-order modes. The resonator and its mirror image excited by (**a**) a single MD emitter, and (**b**) a MD emitter and its mirror image $\text {MD}'$. All the geometrical parameters are the same as Fig. 8. Note that radiated power is normalized to free-space radiation power (**a**) $\text{ P}_0$ and (**b**) $2 \text{ P}_0$. The latter normalization in (**b**) is equivalent to evaluate the ratio of the radiated power by a single emitter in the half space above the PEC and the total radiated power $\text{ P}_0$ in free space.

To provide a full picture in the origin of the high-*Q*-factor resonance excited in the nanoresonator-on-PEC system, we evaluate the normalized radiated power provided by a dimer formed by our dielectric nanoresonator and its mirror image. In our analysis we include the individual contribution of different multipolar terms of the radiated fields, as shown in Fig. 9. We examine two distinct configurations: (i) a PEC-induced dimer excited by a single MD emitter set in the axis of symmetry of the structure, shown in Fig. 9a, and (ii) the dielectric dimer simultaneously excited by two coherent out-of-phase magnetic emitters, coined as MD and $\text {MD}'$ in Fig. 9b. A single MD emitter excites both MD and MQ modes of the silicon dimer at wavelengths $\lambda =602$ nm and $\lambda =561$ nm, respectively, as illustrated Fig. 9a. Though the MQ mode is dominant in the radiation spectrum, which is characterized by a narrowband resonance revealing its high *Q* factor, the contribution of the MD mode is far from negligible. On the other hand, the presence of the image $\text {MD}'$ emitter induces a full cancellation of the MD resonance at far-field, whereas the MQ signal is strongly enhanced, as shown in Fig. 9b. This suggests a superposition of two out-of-phase radiating fields driven by the hybridized MD bright mode of the dimer, resulting in an *effective dark mode*. In addition, a constructive interaction of coherent fields is caused by a MQ modal excitation with inherent high *Q* factor.

Interestingly, radiated fields from a resonant nanoscatterer (also a dimer) upon excitation of a MQ mode experience a $\pi$-phase shift when comparing forward- ($z > 0$) and backward-propagating ($z < 0$) waves. An effect of this kind has also been observed in silicon nanospheres on substrates illuminated by a focused azimuthally polarized beam^37^. This suggests an effective mechanism to compensate the out-of-phase interference of the radiation emitted by the point MD source and its PEC image.

## Conclusion

In summary, the magnetic Purcell factor when a quantum emitter is efficiently coupled to a tapered angle well spherical silicon resonator was investigated. Also the spherical shell resonator and the cylindrical resonator with conical hole are occasionally included in our analysis, however demonstrating a lower performance. It was found that the magnetic PF and the resonance wavelength critically depend on the geometry of the photonic nanostructures. Because of the lower loss of silicon at longer wavelengths in the visible, the quantum efficiency of the axi-symmetric resonator is additionally enhanced by MD modes with excitation at low frequencies. For instance, by increasing the size of the holey silicon sphere when immersed in air, the PF peak frequency is red-shifted as expected while the Purcell factor decreases. Also, narrower conical wells are more favourable to enhance the decay rate of the MD emitter, the latter preferably localized near the hole apex. Finally, the effect of the presence of a metallic substrate was examined, as considered of practical interest in the experimental implementation of our proposal. Driven by a strong image-induced magnetic-quadrupolar resonance, the magnetic Purcell factor was significantly enhanced over $10^3$, which is barely twice the PF of an isolated silicon nanosphere. If in addition the resonator is partially embedded within the metallic substrate, further Purcell enhancement can be observed, attributed to the metallization of the resonator walls and its subsequent increase in the *Q* factor of the excited MQ mode.

## Methods

### Theoretical background and design methodology

In this section, we briefly recall two different approaches to analyze the magnetic Purcell effect. Once a quantum emitter is excited to an upper level, it relaxes into its ground state by emitting a photon with energy $\hbar \omega _0$, where $\omega _0$ is the transition angular frequency of the two levels involved. The spontaneous emission rate of the excited atom located in the free space that in addition is characterized by a transition MD moment ${\mathbf {m}}$ is given by^12^:

4 $$\begin{aligned} \gamma _0=\frac{\omega _0^3}{3\pi \hbar c^3} \mu _0|{\mathbf {m}}|^2 . \end{aligned}$$

Here, $\hbar$, $\mu _0$, and *c*, are the reduced Planck constant, permeability and speed of light in vacuum, respectively. In the case the quantum emitter is located in the vicinity of a complex medium, however, its decay rate changes. According to the Fermi’s golden rule, the spontaneous emission rate $\gamma$ of the MD emitter can be written as^12^:

5 $$\begin{aligned} \gamma =\frac{\pi \omega _0\mu _0}{\hbar } |{\mathbf {m}}|^2 \rho _m(r_0,\omega _0), \end{aligned}$$

where $\rho _m(r_0,\omega _0)$ is the magnetic local density of states (LDOS) as follows:

6 $$\begin{aligned} \rho _m(r_0,\omega _0)=\frac{2\omega _0}{\pi c^2}\big [{\mathbf {n}} \cdot \text {Im} \big ({\mathbf {G}}(r_0,r_0,\omega _0)\big ) \cdot {\mathbf {n}} \big ]. \end{aligned}$$

Here, ${\mathbf {G}}(r_0,r_0,\omega _0)$ denotes the dyadic Green’s function of a MD emitter, ${\mathbf {n}}$ is the unit vector pointing along the direction of the transition MD moment. According to Eq. (6), the spontaneous emission rate can be modified through the magnetic LDOS, which is in relation to the local magnetic field intensity of the resonant mode^44^. Therefore, the induced magnetic field in the direction of the MD can significantly alter the decay rate of the emitter.

On the other hand, from a classical point of view, the emission rate of an oscillating MD can be understood as a work done by the oscillating magnetic current. The total power of the MD point source will be determined by means of the normal component of the time-averaged energy flow density ${\mathbf {S}}=\frac{1}{2} \text{ Re }{[{\mathbf {E}} \times {\mathbf {H}}^*]}$ as integrated through a surface runs over the source. Using the Poynting theorem, the power radiated by the MD emitter can equivalently be obtained as^12^:

7 $$\begin{aligned} \text{ P } = \frac{\omega _0}{2} \text {Im}[{\mathbf {m}}^*\cdot {\mathbf {H}}(r_0)] . \end{aligned}$$

Let us point out that here we use concepts of classical electromagnetism. As a consequence, ${\mathbf {m}}$ in Eq. (7) stands for a MD moment, which does not exactly represents the *transition MD moment* used in Eqs. (4)–(6).

The enhancement of the spontaneous emission rate is commonly established by a dimensionless quantity, the Purcell factor $\text{ F}_\text{ P } = {\gamma }/{\gamma _0}$, which represents the ratio between the spontaneous emission rate of the quantum emitter in a given environment to its spontaneous emission rate in free space. Alternatively, the Purcell factor is evaluated as

8 $$\begin{aligned} \text{ F}_\text{ P } = \frac{\text{ P }}{\text{ P}_0} , \end{aligned}$$

where P and $\text{ P}_0=\frac{|{\mathbf {m}}|^2 \mu _0 \omega _0^4}{12 \pi c^3}$ denote the total power radiated by the point MD source in the complex medium and in free space, respectively. It must be mentioned that, under both perspectives, the PF does not depend on the magnitude of the vector ${\mathbf {m}}$ but on its orientation. In the following, we will carry out our numerical calculations based on the latter procedure, which is a time-harmonic scattering problem of classical electromagnetism, assuming a point source of unit MD moment.

When parts of the emitter environment are lossy, the nonradiative power $\text{ P}_\text{ nr }$ that is dissipated in the environment should be taken into account. Therefore, the total power emitted by the source can be split into two parts, $\text{ P }=\text{ P}_\text{ r }+\text{ P}_{\text {nr}}$, where $\text{ P}_\text{ r }$ is the power radiated into the far-field. As a consequence, we define the normalized radiated power

9 $$\begin{aligned} \text{ F}_\text{ r } = \frac{\text{ P}_\text{ r }}{\text{ P}_0} , \end{aligned}$$

which estimates the power enhancement transferred to the far-field. Finally, one may determine the quantum yield,

10 $$\begin{aligned} \eta = \frac{\text{ P}_\text{ r }}{\text{ P }} , \end{aligned}$$

which establishes the fraction of the total emitted power that is radiated to the far-field.

### Computational methods

The numerical simulations are performed by using the commercial software COMSOL Multiphysics (RF module, frequency domain), which is based on the finite element method (FEM)^45^. To calculate $\text{ F}_\text{ P }$ and $\text{ F}_\text{ r }$, in our model, closed surfaces surrounding only the emitter, and the emitter together with the proposed nearby resonator, are traced respectively. Later on, the total flux over the defined surfaces is calculated. The computational domain is circumscribed within a sphere of two wavelengths in radius. A perfectly matched layer (PML) of one-wavelength thickness is implemented to minimize artificial reflections from the domain boundaries. To ensure the required accuracy of the numerical calculations and minimize the computational time, we gradually decreased the mesh element size to obtain comparably mesh-independent results. The size of the mesh is no larger than $\lambda /10$, and 20 nm in the surrounding medium and the resonator, respectively. The maximum mesh element size in the spherical surfaces enclosing the dipole in order to evaluate the Poynting vector flux thus enabling the estimation of the PF (a sphere of radius 2 nm) and the radiated power (a sphere of one-wavelength radius), are chosen as 0.7 nm and $\lambda /12$, respectively.

## Supplementary Information

Supplementary Information 1.

## References

[1] Purcell, E. M. Spontaneous emission probabilities at radio frequencies. In *Confined Electrons and Photons*, 839 (Springer, 1995).

[2] Pelton M (2015). Modified spontaneous emission in nanophotonic structures. Nat. Photon..

[3] Krasnok AE (2015). An antenna model for the Purcell effect. Sci. Rep..

[4] Gu Q (2013). Purcell effect in sub-wavelength semiconductor lasers. Opt. Express.

[5] Aigouy L, Cazé A, Gredin P, Mortier M, Carminati R (2014). Mapping and quantifying electric and magnetic dipole luminescence at the nanoscale. Phys. Rev. Lett..

[6] Michaelis J, Hettich C, Mlynek J, Sandoghdar V (2000). Optical microscopy using a single-molecule light source. Nature.

[7] Frimmer M, Chen Y, Koenderink AF (2011). Scanning emitter lifetime imaging microscopy for spontaneous emission control. Phys. Rev. Lett..

[8] Andres-Penares D (2021). Out-of-plane trion emission in monolayer wse 2 revealed by whispering gallery modes of dielectric microresonators. Commun. Mater..

[9] Ofelt G (1962). Intensities of crystal spectra of rare-earth ions. J. Chem. Phys..

[10] Zurita-Sánchez JR, Novotny L (2002). Multipolar interband absorption in a semiconductor quantum dot: ii: magnetic dipole enhancement. J. Opt. Soc. Am. B.

[11] Caselli N (2021). Near-field imaging of magnetic complex mode volume. ACS Photon..

[12] Baranov DG, Savelev RS, Li SV, Krasnok AE, Alù A (2017). Modifying magnetic dipole spontaneous emission with nanophotonic structures. Laser Photon. Rev..

[13] Mi H, Wang L, Zhang Y, Zhao G, Jiang R (2019). Control of the emission from electric and magnetic dipoles by gold nanocup antennas. Opt. Express.

[14] Yang Y, Zhu BF, Dai HT, Sun XW (2019). Multiband enhancement of magnetic dipole emission with tapered hollow hyperbolic metamaterials. Opt. Express.

[15] Kuznetsov AI (2014). Split-ball resonator as a three-dimensional analogue of planar split-rings. Nat. Commun..

[16] Li J, Verellen N, Van Dorpe P (2017). Enhancing magnetic dipole emission by a nano-doughnut-shaped silicon disk. ACS Photon..

[17] Feng T, Zhang W, Liang Z, Xu Y, Miroshnichenko AE (2018). Isotropic magnetic Purcell effect. ACS Photon..

[18] Yang Y, Zhu B, Dai H, Sun X (2019). Identical emission enhancement for arbitrary-orientation magnetic dipole emitters in silicon hollow nanocavity. Opt. Express.

[19] Aslan, E. Germanium hollow nanodisk resonator for magnetic dipole decay rate enhancement in near-infrared. *Microw. Opt. Technol. Lett.* **63**, 279–285 (2021).

[20] Evlyukhin AB, Reinhardt C, Seidel A, Luk’yanchuk BS, Chichkov BN (2010). Optical response features of Si-nanoparticle arrays. Phys. Rev. B.

[21] García-Etxarri A (2011). Strong magnetic response of submicron silicon particles in the infrared. Opt. Express.

[22] Evlyukhin AB (2012). Demonstration of magnetic dipole resonances of dielectric nanospheres in the visible region. Nano Lett..

[23] Bakker RM (2015). Magnetic and electric hotspots with silicon nanodimers. Nano Lett..

[24] Wu H-W (2019). Strong Purcell effect for terahertz magnetic dipole emission with spoof plasmonic structure. ACS Appl. Nano Mater..

[25] Wu H-W, Quan J-Q, Yin Y-Q, Sheng Z-Q (2020). Strong Purcell effect for magnetic dipole emission with spoof plasmonic spiral structure. J. Opt. Soc. Am. B.

[26] Gramotnev DK, Vogel MW, Stockman MI (2008). Optimized nonadiabatic nanofocusing of plasmons by tapered metal rods. J. Appl. Phys..

[27] Thomas S, Wachter G, Lemell C, Burgdörfer J, Hommelhoff P (2015). Large optical field enhancement for nanotips with large opening angles. New J. Phys..

[28] Aglieri V (2020). Improving nanoscale terahertz field localization by means of sharply tapered resonant nanoantennas. Nanophotonics.

[29] Bozhevolnyi SI (2006). Effective-index modeling of channel plasmon polaritons. Opt. Express.

[30] Zenin VA (2011). Dispersion of strongly confined channel plasmon polariton modes. J. Opt. Soc. Am. B.

[31] Smith CL, Stenger N, Kristensen A, Mortensen NA, Bozhevolnyi SI (2015). Gap and channeled plasmons in tapered grooves: a review. Nanoscale.

[32] Pashaei Adl H (2020). Purcell enhancement and wavelength shift of emitted light by CsPbI$$_3$$ perovskite nanocrystals coupled to hyperbolic metamaterials. ACS Photon..

[33] Karaveli S, Zia R (2011). Spectral tuning by selective enhancement of electric and magnetic dipole emission. Phys. Rev. Lett..

[34] Huang Z (2015). Strong-field-enhanced spectroscopy in silicon nanoparticle electric and magnetic dipole resonance near a metal surface. J. Phys. Chem. C.

[35] Miroshnichenko AE, Evlyukhin AB, Kivshar YS, Chichkov BN (2015). journaltitleSubstrate-induced resonant magnetoelectric effects for dielectric nanoparticles. ACS Photon..

[36] Sinev I (2016). Polarization control over electric and magnetic dipole resonances of dielectric nanoparticles on metallic films. Laser Photon. Rev..

[37] Deng F, Liu H, Lan S (2018). Metal substrate-induced line width compression in the magnetic dipole resonance of a silicon nanosphere illuminated by a focused azimuthally polarized beam. Nanoscale Res. Lett..

[38] Aspnes DE, Studna A (1983). Dielectric functions and optical parameters of Si, Ge, GaP, GaAs, GaSb, InP, InAs, and InSb from 1.5 to 6.0 eV. Phys. Rev. B.

[39] Jackson, J. D. *Classical Electrodynamics* (John Wiley & Sons Inc., 1999).

[40] Grahn P, Shevchenko A, Kaivola M (2012). Electromagnetic multipole theory for optical nanomaterials. New J. Phys..

[41] Feng T, Zhang W, Liang Z, Xu Y (2018). Unidirectional emission in an all-dielectric nanoantenna. J. Phys. Condens. Matter.

[42] Feng T, Xu Y, Liang Z, Zhang W (2016). All-dielectric hollow nanodisk for tailoring magnetic dipole emission. Opt. Lett..

[43] Johnson PB, Christy R-W (1972). Optical constants of the noble metals. Phys. Rev. B.

[44] Choi B, Iwanaga M, Sugimoto Y, Sakoda K, Miyazaki HT (2016). Selective plasmonic enhancement of electric-and magnetic-dipole radiations of Er ions. Nano Lett..

[45] COMSOL. Software for multiphysics simulation. http://www.comsol.com. Accessed: 2021-04-07.

