# Supplementary material for: Multipolar-sensitive engineering of magnetic dipole spontaneous emission with a dielectric nanoresonator antenna
Source: Sci Rep. 2021 Jun 17;11:12813. doi: 10.1038/s41598-021-92322-9 (PMC8211853; doi:10.1038/s41598-021-92322-9)
Supplement: Supplementary file 1 — Supplementary Information 1. [file 41598_2021_92322_MOESM1_ESM.pdf]

Supplementary Information

Multipolar-sensitive engineering of magnetic dipole spontaneous emission with a dielectric nanoresonator antenna

Mojtaba Karimi Habil[1, *], Carlos J. Zapata–Rodríguez[2], Mauro Cuevas[3,4], and Samad Roshan Entezar[1]

[1]*Faculty of Physics, University of Tabriz, 51664, Tabriz, Iran*
[2]*Department of Optics and Optometry and Vision Sciences, University of Valencia, Dr. Moliner 50, Burjassot 46100, Spain*
[3]*Consejo Nacional de Investigaciones Científicas y Técnicas (CONICET), Buenos Aires, Argentina*
[4] *Facultad de Ingeniería, Universidad Austral, Mariano Acosta 1611, Pilar, Buenos Aires, Argentina*

**m.karimi@tabrizu.ac.ir*

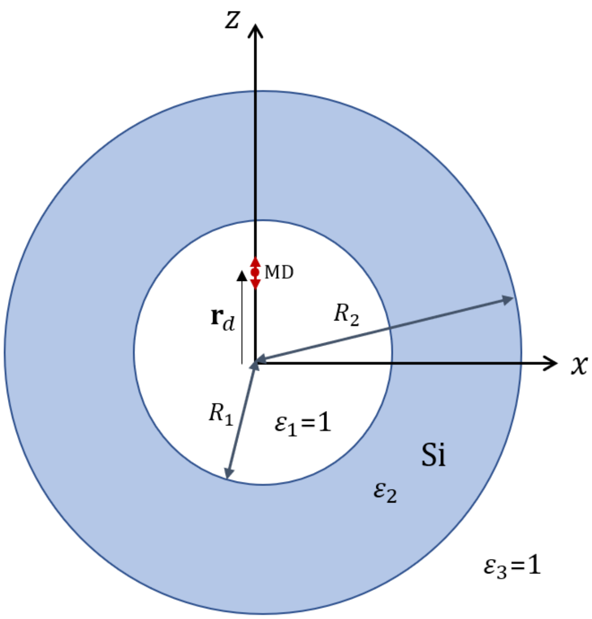

Figure S1. xz-plane intersection of spherical hollow silicon nanocavity suspended in the vacuum. A magnetic dipole oriented in the z direction is placed inside the hollow cavity at the on-axis point $\mathbf{r}_d = r'\hat{z}$.

We consider a sphere consisting in two concentric spherical layers of radius R_1 and R_2 ($R_1 < R_2$), as shown in Fig. S1. The core ($r < R_1$) is vacuum (relative permittivity $\varepsilon_1 = 1$), and shell ($R_1 < r < R_2$) are filled with a dielectric material with relative permittivity ε_2 (silicon in our case). The sphere is embedded in vacuum ($\varepsilon_3 = 1$). Note that all the materials are non–magnetics (relative permeability $\mu_n = 1$, in region $n = 1, 2, 3$). A MD emitter with moment $\mathbf{m}$ is placed at $\mathbf{r}_d = r'\hat{z}$ at a distance $r' < R_1$ from the center of the sphere. In a given region of index n, the fields can be expanded as a series of vector wave functions $\mathbf{M}_{l,m}^{(n,z)}(\mathbf{r})$ and $\mathbf{N}_{l,m}^{(n,z)}(\mathbf{r})$,

$$\mathbf{M}_{l,m}^{(n,z)}(\mathbf{r}) = z_l(k_n r)\mathbf{X}_{l,m}(\hat{\boldsymbol{r}}), \quad (1)$$

$$\mathbf{N}_{l,m}^{(n,z)}(\mathbf{r}) = \frac{i}{k_n}\nabla \times \mathbf{M}_{l,m}^{(n,z)}(\mathbf{r}), \quad (2)$$

where

$$\mathbf{X}_{l,m}(\hat{\boldsymbol{r}}) = \frac{1}{\sqrt{l(l+1)}}\mathbf{L}Y_{l,m}(\hat{\boldsymbol{r}}) \quad (3)$$

is the vector spherical harmonics, $\mathbf{L} = -i\,\mathbf{r} \times \nabla$ is the angular momentum operator, z_l stands for any of the spherical Bessel functions $j_l(x)$ and $y_l(x)$ and the spherical Hankel function $h_l(x)$ of the first kind, l, m are the polar and azimuthal modal numbers denoting the multipolar geometry[1], $Y_{l,m}(\hat{\boldsymbol{r}})$ is a spherical harmonic function of degree l and order m, and $k_n = \sqrt{\varepsilon_n\,\mu_n}\omega/c$ is the wavenumber in the medium of index n.

In region $n = 1$ ($r < R_1$) the EM field is a superposition of two contributions: the electric field $\mathbf{E}_{inc}$ and magnetic field $\mathbf{H}_{inc}$ emitted by the MD, and the scattered fields $\mathbf{E}_1$ and $\mathbf{H}_1$. The incident field emitted by the magnetic dipole is written as

$$\mathbf{H}_{inc}(\mathbf{r}) = \sum_{l,m} a_{l,m}^{(0E>)}\ \mathbf{M}_{l,m}^{(1h)}(\mathbf{r}) - a_{l,m}^{(0M>)}\ \mathbf{N}_{l,m}^{(1h)}(\mathbf{r}), \quad (4)$$

$$\mathbf{E}_{inc}(\mathbf{r}) = Z_0 \sum_{l,m} a_{l,m}^{(0E>)}\ \mathbf{N}_{l,m}^{(1h)}(\mathbf{r}) + a_{l,m}^{(0M>)}\ \mathbf{M}_{l,m}^{(1h)}(\mathbf{r}), \quad (5)$$

for $r' < r < R_1$ and

$$\mathbf{H}_{inc}(\mathbf{r}) = \sum_{l,m} a_{l,m}^{(0E<)}\ \mathbf{M}_{l,m}^{(1j)}(\mathbf{r}) - a_{l,m}^{(0M<)}\ \mathbf{N}_{l,m}^{(1j)}(\mathbf{r}), \quad (6)$$

$$\mathbf{E}_{inc}(\mathbf{r}) = Z_0 \sum_{l,m} a_{l,m}^{(0E<)}\ \mathbf{N}_{l,m}^{(1j)}(\mathbf{r}) + a_{l,m}^{(0M<)}\ \mathbf{M}_{l,m}^{(1j)}(\mathbf{r}), \quad (7)$$

for $r < r'$, where $Z_0 = \sqrt{\mu_0/\varepsilon_0}$ is the vacuum impedance (ε_0, μ_0 the vacuum permittivity and permeability, respectively). The first term in Eqs. (4) and (6) represents the transverse contribution to the magnetic field (TM polarization) while the second components represents the transverse contribution to the electric field (TE polarization). The complex amplitudes $a_{l,m}^{(0E>)}$, and $a_{l,m}^{(0M>)}$ are

$$a_{l,m}^{(0E>)} = -\frac{4\pi\omega^2 k_1}{c^2\sqrt{l(l+1)}}\, i j_l(k_1 r')\mathbf{m} \cdot \mathbf{L}' Y_{lm}^*(\hat{\boldsymbol{r}}'), \quad (8)$$

$$a_{l,m}^{(0M>)} = \frac{4\pi\omega^2}{c^2\sqrt{l(l+1)}}\mathbf{m} \cdot \nabla' \times [j_l(k_1 r')\mathbf{L}' Y_{lm}^*(\hat{\boldsymbol{r}}')], \quad (9)$$

where $\hat{\boldsymbol{r}}' = \mathbf{r}_d/r'$ (here $\hat{z}$), while the expressions for $a_{l,m}^{(0E<)}$ and $a_{l,m}^{(0M<)}$ are obtained from Eqs. (8) and (9), respectively, by changing $j_l(x) \leftrightarrow h_l(x)$.

The scattered fields in region 1 are written as

$$\mathbf{H}_1(\mathbf{r}) = \sum_{l,m} a_{l,m}^{(1E)}\ \mathbf{M}_{l,m}^{(1j)}(\mathbf{r}) - a_{l,m}^{(1M)}\ \mathbf{N}_{l,m}^{(1h)}(\mathbf{r}), \quad (10)$$

$$\mathbf{E}_1(\mathbf{r}) = Z_0 \sum_{l,m} a_{l,m}^{(1E)}\ \mathbf{N}_{l,m}^{(1h)}(\mathbf{r}) + a_{l,m}^{(1M)}\ \mathbf{M}_{l,m}^{(1h)}(\mathbf{r}). \quad (11)$$

In the other regions, the EM field is written as,

$$\mathbf{H}_2(\mathbf{r}) = \sum_{l,m} a_{l,m}^{(2Ej)}\ \mathbf{M}_{l,m}^{(2j)}(\mathbf{r}) - a_{l,m}^{(2Mj)}\ \mathbf{N}_{l,m}^{(2j)}(\mathbf{r}) + a_{l,m}^{(2Ej)}\ \mathbf{M}_{l,m}^{(2y)}(\mathbf{r}) - a_{l,m}^{(2My)}\ \mathbf{N}_{l,m}^{(2y)}(\mathbf{r}), \quad (12)$$

$$\mathbf{E}_2(\mathbf{r}) = Z_2 \sum_{l,m} a_{l,m}^{(2Ej)}\ \mathbf{N}_{l,m}^{(2j)}(\mathbf{r}) + a_{l,m}^{(2Mj)}\ \mathbf{M}_{l,m}^{(2j)}(\mathbf{r})$$

[1] do not confuse m with the modulus of the MD moment $\mathbf{m}$

$$+a_{l,m}^{(2Ej)}\ \mathbf{N}_{l,m}^{(2y)}(\mathbf{r}) + a_{l,m}^{(2My)}\ \mathbf{M}_{l,m}^{(2y)}(\mathbf{r}), \tag{13}$$

for $R_1 < r < R_2$, $Z_2 = Z_0\sqrt{\mu_2/\varepsilon_2}$ and

$$\mathbf{H}_3(\mathbf{r}) = \sum_{l,m} a_{l,m}^{(3E)}\ \mathbf{M}_{l,m}^{(3h)}(\mathbf{r}) - a_{l,m}^{(3E)}\ \mathbf{N}_{l,m}^{(3h)}(\mathbf{r}), \tag{14}$$

$$\mathbf{E}_3(\mathbf{r}) = Z_0 \sum_{l,m} a_{l,m}^{(3E)}\ \mathbf{N}_{l,m}^{(3h)}(\mathbf{r}) + a_{l,m}^{(3M)}\ \mathbf{M}_{l,m}^{(3h)}(\mathbf{r}), \tag{15}$$

for $r > R_3$.

At this point, it is interesting to note that Eqs. (1) and (2) in the main text can be derived by multiplying Eqs. (14) and (15) by **r** and integrating throughout the solid angle.

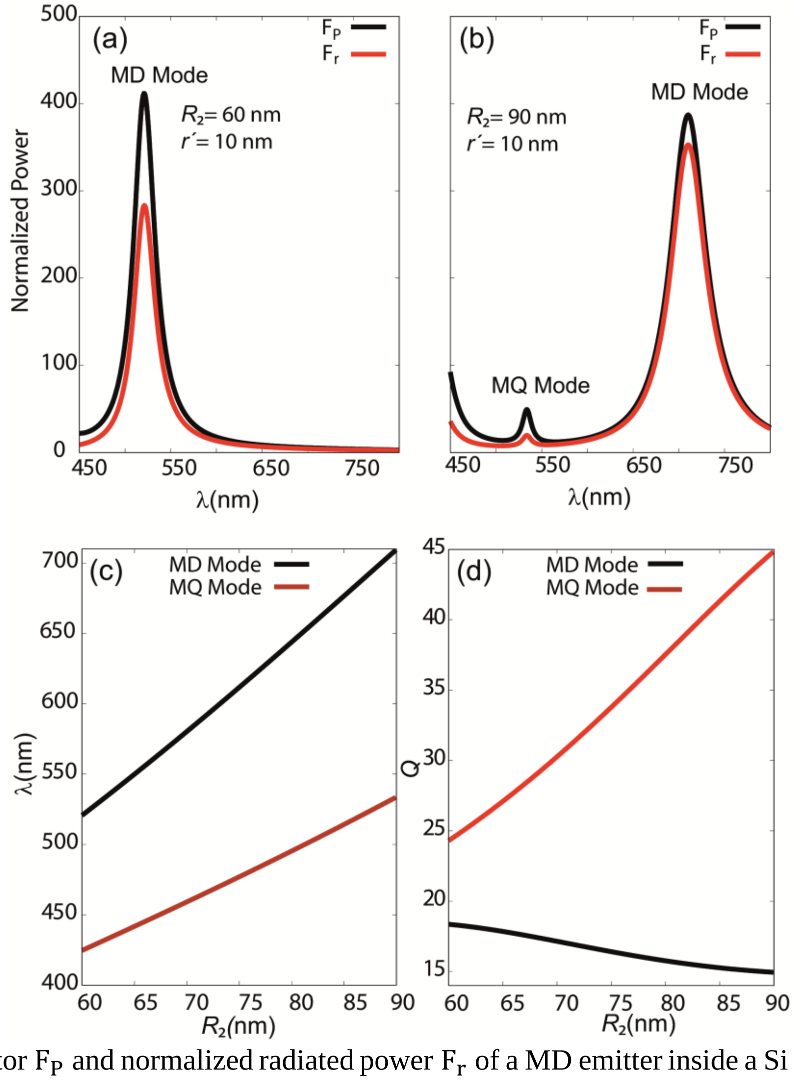

Figure S2. Purcell factor F_P and normalized radiated power F_r of a MD emitter inside a Si shell with inner radius $R_1 = 20$ nm and outer radius (a) $R_2 = 60$ nm and (b) $R_2 = 90$ nm. The dipole is 10 nm from the sphere center. (c) The eigenwavelength $\lambda = 2\pi c/Re(\omega)$ for the first two magnetic modes, MD ($l = 1$) and MQ ($l = 2$), calculated by solving the complex dispersion equation $1/R_l^{TE}(\omega) = 0$, as a function of the outer radius R_2 while keeping the inner radius $R_1 = 20$ nm. We observe that λ values match very well with the spectral peaks in (a) and (b). (d) The quality factor $Q = -Re(\omega)/2Im(\omega)$ is a decreasing function of R_2 for the MD eigenmode and an increasing function for the MQ eigenmode.

By imposing the well-known boundary conditions on interfaces $r = R_1$ and $r = R_2$, *i.e.*, letting

the tangential component of the total electric **E** and magnetic **H** fields to be continuous on the surface separating two different media, we find the complex amplitudes $a_{l,m}^{(1E)}$, $a_{l,m}^{(1M)}$, $a_{l,m}^{(2Ej)}$, $a_{l,m}^{(2Mj)}$, $a_{l,m}^{(2Ey)}$, $a_{l,m}^{(2My)}$, $a_{l,m}^{(3E)}$ and $a_{l,m}^{(3M)}$. In particular, in region 1 the amplitudes $a_{l,m}^{(1E)} = R_l^{TM}\, a_{l,m}^{(0E>)}$, $a_{l,m}^{(1M)} = R_l^{TE}\, a_{l,m}^{(0M>)}$, while in region 3 the amplitudes $a_{l,m}^{(3E)} = T_l^{TM}\, a_{l,m}^{(0E>)}$, $a_{l,m}^{(3M)} = T_l^{TE}\, a_{l,m}^{(0M>)}$, where $R_l^{TM}, R_l^{TE}, T_l^{TM}, T_l^{TE}$ are the modal reflection and transmission coefficients for both polarizations [1].

By taking into account the radial polarization of the magnetic dipole moment, $\mathbf{m} \parallel \hat{r}$, from Eqs. (8) and (9), we arrive to $a_{l,m}^{(0E>)} = a_{l,m}^{(0E<)} = 0$, that is the fields are purely TE-polarized. In addition,

$$a_{l,m}^{(0M>)} = \frac{4\pi\omega^2}{c^2\sqrt{l(l+1)}} i|\mathbf{m}| \frac{l(l+1)}{r'} j_l(k_1 r') Y_{l,m}^*(\hat{\boldsymbol{r}}') = \sqrt{4\pi}\frac{\omega^2}{c^2}\sqrt{l(l+1)(2l+1)}\, i|\mathbf{m}| \frac{j_l(k_1 r')}{r'}\delta_{m,0}, \tag{16}$$

and

$$a_{l,m}^{(0M<)} = \frac{4\pi\omega^2}{c^2\sqrt{l(l+1)}} i|\mathbf{m}| \frac{l(l+1)}{r'} h_l(k_1 r') Y_{l,m}^*(\hat{\boldsymbol{r}}') = \sqrt{4\pi}\frac{\omega^2}{c^2}\sqrt{l(l+1)(2l+1)}\, i|\mathbf{m}| \frac{h_l(k_1 r')}{r'}\delta_{m,0}, \tag{17}$$

where in the last equality we have used

$$Y_{l,m}^*(\hat{\boldsymbol{r}}') = Y_{l,m}^*(\theta' = 0, \phi' = 0) = \sqrt{\frac{(2l+1)}{4\pi}}\delta_{m,0}.$$

As a consequence, only azimuthally-symmetric fields with $m = 0$ are excited in the optical resonator.

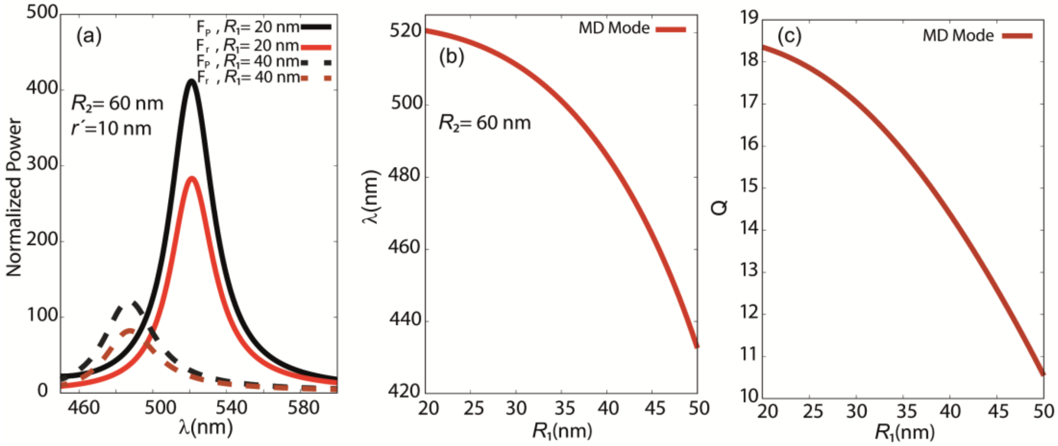

Figure S3. (a) Purcell factor F_P and normalized radiated power F_r of a MD emitter inside a Si spherical shell for $R_1 = 20$ nm and $R_1 = 40$ nm, keeping $R_2 = 60$ nm. (b) The eigenwavelength corresponding to the MD mode of the shell resonator, which shows a blue-shift by the radius increment of the inner hole. (c) The Q factor for the MD eigenmode, supporting a drop of the PF as the volume of the vacuum hole is increased.

Once the total EM field is known, we can calculate the Purcell factor F_P and the normalized power F_r radiated into the medium $n = 3$ as follows. The time–averaged emitted power P is calculated from the integral of the radial component of the Poynting vector flux through an imaginary sphere

of radius $\rho_0 \rightarrow 0$ that encloses the magnetic dipole, giving

$$\mathrm{F_P} = \frac{\mathrm{P}}{\mathrm{P_0}} = 1 + \frac{3}{2}\sum_l l(l+1)(2l+1)\mathrm{Re}\left\{R_l^{TE}\left[\frac{j_l(k_1 r\prime)}{k_1 r\prime}\right]^2\right\}, \tag{18}$$

where

$$\mathrm{P_0} = \frac{|\mathbf{m}|^2 \mu_0 \omega^4}{12\pi c^3},$$

is the power emitted by the same MD embedded in the (ideally) unbounded medium $n = 1$. On the other hand, the time–averaged scattered power $\mathrm{P_r}$ is calculated from the integral of the radial component of the complex Poynting vector flux through an imaginary sphere of radius $\rho_0 > R_2$, providing

$$\mathrm{F_r} = \frac{\mathrm{P_r}}{\mathrm{P_0}} = \frac{3}{2}\sum_l l(l+1)(2l+1)\left|T_l^{TE}\frac{j_l(k_1 r\prime)}{k_1 r\prime}\right|^2. \tag{19}$$

Application of Eqs. (18) and (19) to our silicon shell nanoscatterer is shown in Figs. S2 and S3.

References

[1] Chew W C 1990. Waves and Fields in Inhomogeneous Media. New York: Van Nostrand Reinhold (New York: Van Nostrand Reinhold).
